# Bile acids as biomarkers in carbonized archaeological sediment: Insights from dung burning experiments

**DOI:** 10.1371/journal.pone.0312699

**Published:** 2025-02-21

**Authors:** Basira Mir-Makhamad, Thomas Larsen, Daniel Giddings Vassao, Robert Spengler, Yiming V. Wang

**Affiliations:** 1 Department of Archaeology, Max Planck Institute for Geoanthropology, Jena, Thuringia, Germany; 2 Department of Archaeology, Domestication and Anthropogenic Evolution Research Group, Max Planck Institute for Geoanthropology, Jena, Thuringia, Germany; 3 Department of Oriental Studies, Friedrich Schiller University, Jena, Germany; 4 Institue of Geosciences, Faculty of Chemistry and Earth Science, Friedrich Schiller University, Jena, Germany; South China Agricultural University, CHINA

## Abstract

Bile acids are increasingly used as fecal biomarkers for studying archeology, environmental pollution, paleoeconomy, and human-animal interactions. Exclusively synthesized by vertebrates, bile acids are more resistant to diagenetic degradation than other steroidal biomarkers. Although bile acids have been detected and analyzed in archaeological sediments, particularly in contexts where dung may have been used as fuel, their preservation after burning is poorly understood. In this study, we conducted controlled experiments on modern cattle dung to investigate the tolerance of bile acid to high temperatures (125°C, 233°C, 341°C, and 449°C). Bile acids were quantified before and after burning via High-Performance Liquid Chromatography coupled with Electrospray Ionization-Mass Spectrometry (HPLC-EI-MS). Our results indicate that elevated temperatures destabilize most bile acids to varying degrees. Primary and secondary bile acids showed moderate heat tolerance, persisting at reduced concentrations after exposure to the maximum furnace and open-air temperatures. In contrast, oxo-bile acids exhibited lower thermal stability and disappeared at exposure above 233°C. Open-air fires led to more significant overall bile acid loss than the furnace conditions, likely due to the higher temperatures. However, incompletely burned dung fragments from the cooler periphery of the fire pits, retained higher bile acid concentrations than fully combusted ashes. Our findings suggest that high temperatures complicate the use of bile acid profiles to distinguish species of origin. These persistent bile acids can, in archaeological contexts, provide valuable insights into past resource utilization, seasonal fuel use, mobility patterns, and environmental reconstruction.

## Introduction

Humans forged mutualistic relationships with herd animals roughly ten millennia ago, and agropastoralist practices were so successful that a demographic wave brought the descendants of these sheep, goats, and cattle across two continents. While everyone recognizes the significance of meat, wool, hair, blood, bones, skin, and dairy as resources in pastoralist economies, few scholars acknowledge the paramount importance of dung, especially as fuel, for the success of this cultural adaptation [1]. Dung permitted ancient humans into some of the most extreme ecosystems on the planet, including hyper-arid deserts and frozen tundra or high elevations [1]. For example, it has been hypothesized that humans could never have adapted to the Tibetan Plateau without dung, as there were no alternative fuel sources for warmth and cooking [2]. Historically and in many regions today, dung remains the primary fuel source. The increased recognition of its significance has led archaeological researchers to employ various biomolecular techniques to infer the sources of dung and how it was used in the past [3–5]. However, researchers face significant challenges in interpreting biochemical profiles from burned dung remains, as the combustion process can alter the chemical composition and structure of fecal biomarkers. This degradation may limit their use for interpreting ancient livestock management and domestication practices.

Fecal biomarkers, including Δ^5^-sterols, stanols, and bile acids, can elucidate various aspects of past human-animal-enviornment interactions, such as tracing fecal pollution, tracking the evolution of domestication traits, understanding animal management practices, and reconstructing husbandry practices [4, 6–9]. Among these biomarkers, bile acids are exclusively produced by vertebrates and stand out for their greater resistance to degradation compared to Δ^5^-sterols and stanols. Their preservation potential and source specificity make bile acids a promising candidate biomarker for tracing fecal sources in archaeological sediments [3, 10–12]. However, despite their potential, their long-term preservation remains poorly understood.

Bile acids are steroidal acids produced in the digestive system of vertebrates [7]. They fall into three main categories: primary, secondary, and oxo (or keto). Primary bile acids, such as cholic (CA) and chenodeoxycholic acids (CDCA), are synthesized in the liver as part of the cholesterol metabolic process and released into the intestine through bile. These primary bile acids are extensively transformed by the gut microbiota, giving rise to secondary bile acids like lithocholic (LCA), deoxycholic (DCA), hyodeoxycholic (HDCA), and ursodeoxycholic (UDCA) acids. Only a minimal portion of the secondary bile acids is excreted in feces; the majority, up to 95%, are reabsorbed in the intestine during the enterohepatic cycle, returning to the liver [6, 7, 13]. Together with primary bile acids, up to 10% of the total bile acid pool is released into the environment [14].

Oxo-bile acids are derivatives formed by bacterial oxidation of hydroxyl (-OH) groups on primary and secondary bile acids during intestinal transit [15]. These compounds can account for up to 30% of the total bile acid pool in the gut [16]. While most studies have focused on analyzing oxo-bile acids in human feces [16], Porru et al. [17] were the first to comprehensively investigate the recovery of oxo-bile acids in animal feces. Historically, the recovery and detection of oxo-bile acids have been challenging, with various artefactual products tentatively identified by gas chromatography [18]. Furthermore, oxo-bile acids are particularly susceptible to alkaline hydrolysis [18], which can affect their preservation during extraction procedures, i.e., exposure to different saponification solvents. For example, using water as a saponification medium helps minimize alkaline hydrolysis, which targets the free hydroxyl groups on the steroid skeleton [18]. However, recent advancements in liquid chromatography-mass spectrometry (LC-MS) techniques have reduced the need for harsh pretreatment steps and enabled the detection of a wider range of oxo-bile acid types, making it the preferred analytical approach today [19].

Using bile acids in archaeological studies poses challenges beyond their low release into the environment. Context is crucial, as sediment samples are often collected from variable locations, such as open areas, room fills, middens, and burial sites [11]. However, hearths, fireplaces, and ashy deposits have received less attention. Bull et al. [7] suggested that bile acid preservation is favorable in desiccated environments. Recent research by Vallejo et al. [20], who analyzed bile acids in prehistoric sheepfold caves in Spain, found low preservation of bile acids in most burned layers but a high concentration in one burned sample, likely due to lower thermal alteration. Nevertheless, bile acid’s tolerance to high temperatures and the feasibility to trace it after burning has not yet been systematically studied. Experimental research examining the preservation and concentration of fecal biomarkers under controlled burning conditions are lacking. To fill this knowledge gap, we conducted a dung-burning experiment to assess the preservation potential of bile acid profiles from burned dung and investigate the differential resistance of bile acid types to high temperatures. This study aims to evaluate bile acid tolerance across a gradient of increasing temperatures and determine the feasibility of detecting bile acids in archaeological deposits containing carbonized materials. Building upon the findings of Vallejo et al. [20], we hypothesize that the high temperatures during burning will lead to substantial but varying degradation of bile acids, limiting their preservation and detection in archaeological hearths and ash deposits. We anticipate that oxo-bile acids would exhibit lower thermal stability than the primary and secondary bile acids due to oxo (= O) groups, known as ketone or carbonyl groups, which are generally more susceptible to heat than hydroxyl groups.

## Materials and methods

We collected twenty large dung patties (approximately 20–25 cm in diameter) from freely grazing cattle from a pasture in Ammerbach, Jena, Germany (50.901053⁰N, 11.551643⁰E) in September 2023. To facilitate drying, each paddy was quartered and placed in perforated cardboard boxes under ventilated, shaded conditions at ambient temperature (12–22⁰C) for four weeks before further processing. Dung patties were checked and re-arranged every two days to ensure no mold and microbes growth. Once fully dry, two 5-gram samples were collected as control material and freeze-dried at the Institute of Geosciences, Friedrich Schiller University, Germany. The remaining dried cattle dung was subjected to two different dung-burning experiments to investigate how combustion affects bile acid concentrations. These experiments were conducted in two distinct settings: one simulating real-life dung burning and another using a closed muffle furnace to provide a controlled environment.

### Open-air burning experiment

**Fig 1:**
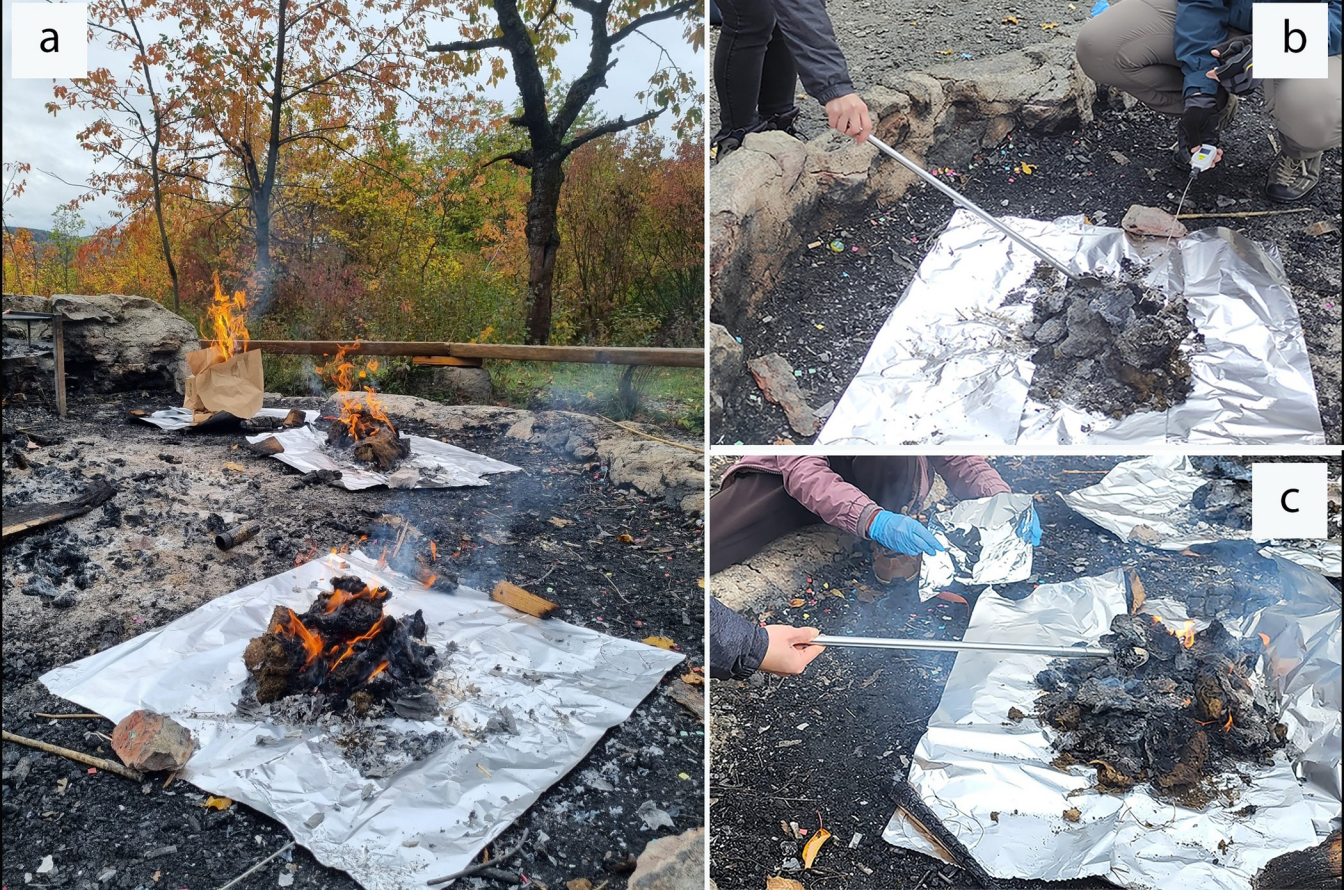
Open fire experiment: **a**—starting three discrete fires with paper bags full of dry dung, **b**–temperature recording every 15 minutes, and **c**–sampling every 15 minutes in an aluminized bag.

The open-air burning experiment was conducted at a designated campfire site in Jena (Fig 1). Three dung piles, each weighing 1 kg, were burned as triplicate samples. Preliminary burning tests estimated that it takes approximately 45 minutes to completely burn 1 kg of dry dung. Therefore, during the burning experiment, subsamples were collected from each pile every 15 minutes, with temperatures recorded at each interval until the burning was completed at 45 minutes. After 45 minutes, most of the dung had turned to ash. However, while pile 2 was completely consumed, piles 1 and 3 still contained small, unburned fragments around the periphery of the fire, where the temperature was cooler. These remnants were also collected for further analysis.

### A closed muffle furnace burning experiment

The muffle furnace burning experiment was conducted at the Institute of Geosciences at Friedrich Schiller University, Jena, to investigate how varying burning temperatures within the same time frame affect bile acid (BA) concentration and preservation. Four sets of dung piles were prepared for this experiment, each consisting of triplicate samples of 50 g dry dung. The first set of samples was subjected to a temperature of 125°C, gradually reached over 60 minutes. Similarly, the second set was heated to a final temperature of 233°C within the same timeframe. The third and fourth sets were subjected to 341°C and 449°C, respectively. The temperature gradient was selected based on the thermal characteristics of deoxycholic acid (DCA). According to a previous study [21], this bile salt mass sharply decreases at a temperature of 450°C. Based on this, we chose four temperature intervals between room temperature to 449°C to track concentration change before a significant mass loss would occur.

After two burning experiments, all samples–including the control /reference, open-air burning, and muffle furnace burning samples–were individually homogenized into a fine powder for further analysis.

### Determination of Total Organic Carbon (TOC)

To monitor the level of organic compounds present in the dry dung and standardize the bile acid content of each sample against the organic matter of each dung sample, we measured Total Organic Carbon (TOC) using loss on ignition method. Two subsamples of air-dried dung, each weighing 3 g, were placed in the muffle furnace at 110°C for two hours to remove residual moisture. After drying, both samples were weighed again and were found to have lost 13.3% of their weight (0.4 g). These samples were subsequently burned at 550°C for two hours [22]. After burning, sample A lost 73.3% of its mass, while sample B lost 76.7%, resulting average TOC of 75%. Both samples were then placed in the oven again at 550°C for an additional two hours, but no further weight changes were observed (Table 1). The TOC of each sample was calculated by multiplying the sample’s mass by the average TOC percentage.

**Table 1:** Total Organic Carbon measurements.

	Sample A (g)	Sample B (g)
Original weight	3.0	3.0
Drying in the oven– 2 hours, 110°C	2.6	2.6
Burning in the oven– 2 hours, 550°C	0.8	0.7
Burning in the oven–(additional 2 hours, 550°C)	0.8	0.7

### Standard selection

The standards used to identify all the bile acids for this work are listed in Table 2. The primary and secondary bile acid standards, including cholic acid (CA), chenodeoxycholic acid (CDCA), hyodeoxycholic acid (HDCA), deoxycholic acid (DCA), and lithocholic acid (LCA), were purchased from Sigma Aldrich (Munich, Germany). The oxo- bile acid standards dehydrocholic acid (trioxo-CA), 3,6-diO-cholanoic acid (3,6-dioxo-HDCA), 3,7-diO-cholanoic acid (3,7-dioxo-CDCA), 3,12-diO-cholanoic acid (3,12-dioxo-DCA), 7α-OH-3-O-cholanoic acid (3-oxo-CDCA), 12α-OH-3-O-cholanoic acid (3-oxo-DCA), 3α-OH-12-O-cholanoic acid (12-oxo-DCA), 3α,12α-diOH-7-O-cholanoic acid (7-oxo-CA), 3α,7α-diOH-12-O-cholanoic acid (12-oxo-CA), 3α,6α-diOH-7-O-cholanoic acid (7-oxo-HCA) were purchased from Steraloids (Newport, USA).

**Table 2:** Bile acids standards used in this study.

Type	Compound	Abbreviations	Ret. Time (min)
Primary acid	Cholic acid	CA	17.25
	Chenodeoxycholic acid	CDCA	26.01
Secondary acid	Deoxycholic acid	DCA	26.81
	Hyodeoxycholic acid	HDCA	19.72
	Lithocholic acid	LCA	38.81
Oxo-bile acid	3,7-diO-Cholanoic acid	3,7-dioxo-CDCA	25.22
	3,6-diO-Cholanoic acid	3,6-dioxo-HDCA	25.66
	3,12-diO-Cholanoic acid	3,12-dioxo-DCA	24.73
	7α-OH-3-O-Cholanoic acid	3-oxo-CDCA	28.66
	12α-OH-3-O-Cholanoic acid	3-oxo-DCA	27.96
	3α-OH-12-O-Cholanoic acid	12-oxo-DCA	22.97
	Dehydrocholic acid	DHCA/Trioxo-CA	14.77
	3α12α-diOH-7-O-Cholanoic acid	7-oxo-CA	14.68
	3α,6α-diOH-7-O-Cholanoic acid	7-oxo-HCA	16.39
	3α,7α-diOH-12-O-Cholanoic acid	12-oxo-CA	14.73
Internal standards for the HPLC	Cholic acid-d4	CA-d4	17.21
	Lithocholic acid-d4	LCA-d4	38.77
	Deoxycholic acid-d4	DCA-d4	26.73
	Chenodeoxycholic acid-d4	CDCA-d4	25.95

In addition to these standards, the deuterated BAs lithocholic acid-d4, deoxycholic acid-d4, cholic acid-d4, and isodeoxycholic acid-d4 were used as internal standards (IS) for the HPLC-ESI-MS/MS analyses. The concentration of stock solution of each standard was 2 mg/mL and was diluted to 20 μg/mL before adding at the beginning of the extraction procedure to each sample to calculate the extraction yield.

### Sample preparation: Extraction

**Fig 2:**
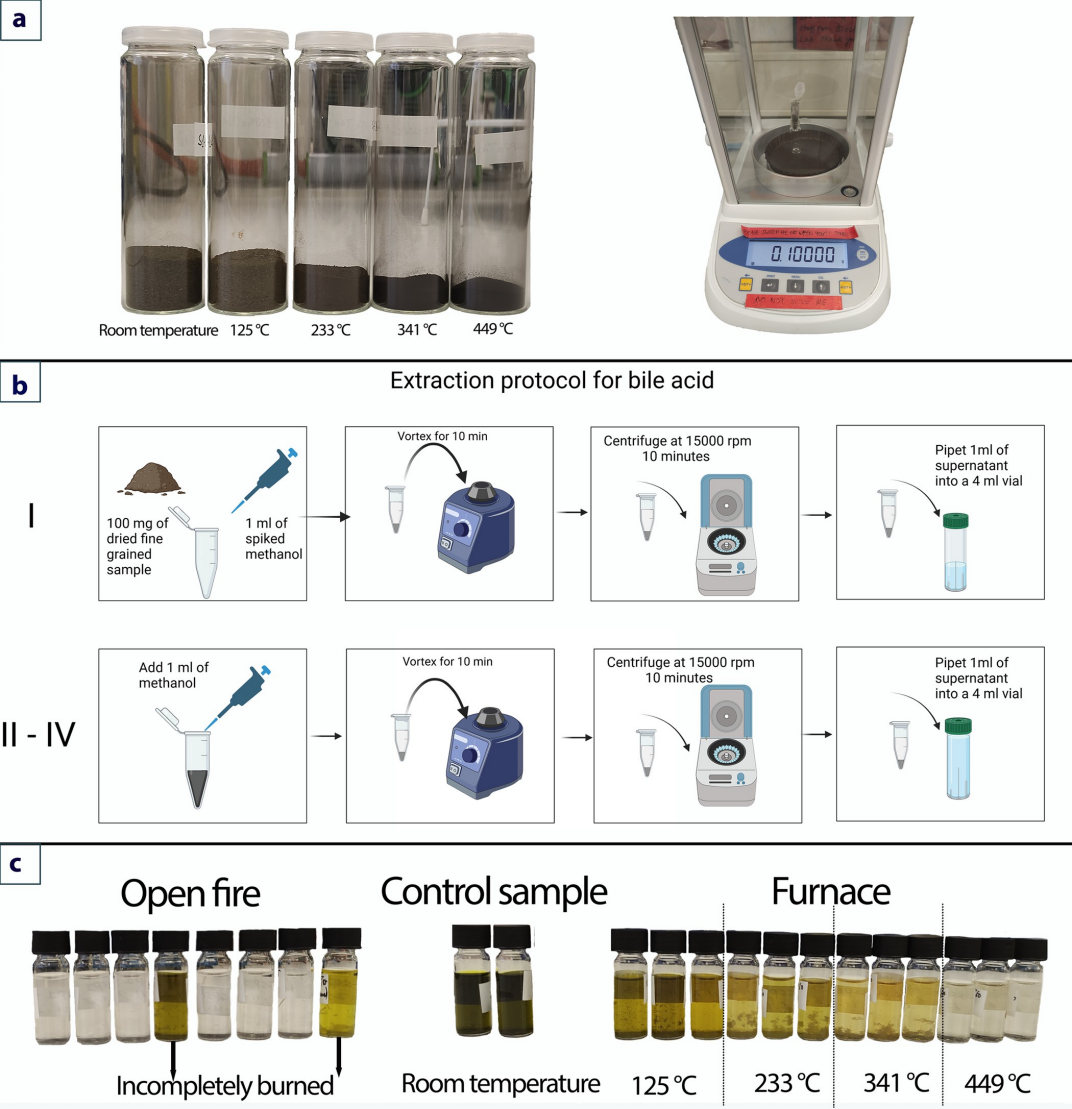
Furnace dung-burning experiment and laboratory pre-treatment: **a**–furnace burning–sample volume decrease control and subsampling of 100 mg of each sample, **b**–bile acids extraction protocol, and **c**–color change of extracted sample depending on the burning temperature.

Approximately 100 mg of each dried sample were weighed and placed into a 1.5 mL Eppendorf vial. For the initial extraction, 1 mL of methanol spiked with internal standards (lithocholic acid-d4, deoxycholic acid-d4, cholic acid-d4, and chenodeoxycholic acid-d4) was added to each vial. Each sample was then vortexed for 10 minutes to ensure thorough mixing. After vortexing, the samples were centrifuged at 15,000 rpm for 10 minutes. The resulting supernatant was directly transferred into a 4 mL dram vial using a Pasteur pipette. Subsequently, the fecal samples were resuspended in 1 mL of methanol (lacking internal standards), vortexed for 10 minutes, and centrifuged for 10 minutes. The supernatant was then pipetted and transferred into the same dram vial. This extraction step was repeated twice more, resulting in four extractions per sample and yielding a combined volume of 4 mL of extract (Fig 2). The decision to limit the number of extractions to four was informed by a preliminary experiment, which indicated an average recovery of 95% for all bile acids after four extractions, as detailed in S1 File. The extract was evaporated to dryness under a gentle stream of nitrogen. After evaporation, samples were redissolved in 300 μL of methanol. Subsequently, 20 μL of each solution was transferred to 1,5 mL GC vials, and 2 μL was injected into the HPLC-ESI-MS.

### Analytical method

HPLC-ESI-MS/MS analyses were conducted using a Shimadzu LCMS-8050 triple-quadrupole MS system. Chromatographic separation was performed on a Shimadzu Shimpack Velox SP-C18 column (100 mm × 2.1 mm, 2.7 μm particle size). The binary solvent system consisted of mobile phases A (HPLC-grade H_2_O with 0.1% formic acid) and B (acetonitrile). The column temperature was fixed at 25°C, with a flow of 0.2 mL/min and the following gradient program: hold 0.5% B from 0–1 min, then rise linearly to 35% B at 10 min, to 48% B at 30 min, to 75% at 40 min, and 100% B at 45 min with hold until 47.5 min, and back to 0.5% B and re-equilibration until 50 min. Ionization was performed with an electrospray ionization (ESI) ion source with detection in negative mode. As in Franco et al. [16], MRM transitions for the different compounds were set, with Q1 and Q3 masses being those of the deprotonated molecules [M–H]^–^, i.e., without fragmentation. Peak identification was carried out based on comparison with retention times of authentic standards where available. LC-MS/MS data were collected and processed using the LabSolutions software (Shimadzu, Kyoto, Japan).

Bile acid quantification was performed using an external calibration curve. The stock bile acids standard solution in methanol was diluted to various concentrations (0.25, 0.5, 1.0, 2.5, 5.0, 10.0, 15.0, and 20.0 μg/mL). The bile acid standards of these varying concentrations were run three times to ensure reproducibility. A calibration curve of the peak area versus concentration was generated for each compound. Since no bile acids were detected in our blanks, we assume all calibration curves began at (0,0) to prevent negative calibration results. We used a linear regression model to calculate the sample abundance. Linearity was considered satisfactory if r^2^ coefficients were more than 0.97 (ranging between 0.97–1). The retention times for each bile acid, listed in Table 2, were determined by calculating the average time from three separate runs of bile acid standards.

### Statistical analyses

All statistical analyses were performed in R version 4.3.2 [23] with RStudio interface version 2023.09.0.463 [24]. We first applied Pillai’s trace Multivariate Analysis of Variance (MANOVA) to test the null hypothesis that there is no significant difference in bile acid concentrations at different temperature intervals. We then performed a univariate analysis of variance analyses (ANOVA) on the MANOVA output to assess which bile acids are less heat resistant and which are relatively more tolerant to high temperatures. We did not perform a MANOVA test on the open fire experiment as we could not control the burning temperature. Since the temperature in an open fire depends on factors such as ventilation at the burning location, uneven heat distribution etc, achieving consistent heat levels was unfeasible Unless otherwise stated, statistical significance was assessed at p < 0.05.

## Results

### Internal standards

The final concentration of four isotope-labeled internal standards from each sample was calculated to assess the mass loss during extraction. The recovery of the internal standards across all samples is fairly consistent, and the average recovery and standard deviation for LCA-d4 are 66±7%; for DCA-d4–45±2%; for CDCA-d4–64±6%; for CA-d4–33±3%. The highest concentration recovery was observed for LCA-d4, while the lowest was for CA-d4. Due to the low recovery of CA-d4, the concentration of CA is likely underestimated compared to the other three internal standards. Since isotope-labeled bile acids were unavailable for each analyzed compound, we did not correct the bile acid concentrations based on internal standard recoveries. This approach aligns with our objective: to examine bile acid preservation and the relative changes in bile acid levels under different burning conditions rather than quantifying absolute concentrations.

### Color change

Before the LC analysis, we observed color variations in the extracts, which differed based on burning temperatures but not the burning duration (Fig 2C). The non-combusted samples are the darkest. In the open-fire burning experiment, the bile acid extracts from samples at the 15-minute interval were colorless, likely due to the high temperatures (up to 740⁰C). Two incompletely burned samples collected at the periphery of fire at the end of the experiment (after 45 minutes of burning), where the temperatures were at 77 ⁰C and 139 ⁰C, respectively, had a greenish color compared to the entirely burned samples. In contrast, extractions from the muffle furnace exhibited a gradual color change that inversely correlated with the temperature. The greenish hue became lighter and less saturated as the temperature increased from 125°C to 449°C, with the extract being completely colorless at 449⁰C. These observations suggest that the temperature was the primary factor influencing the color change.

### Control samples analysis

**Fig 3:**
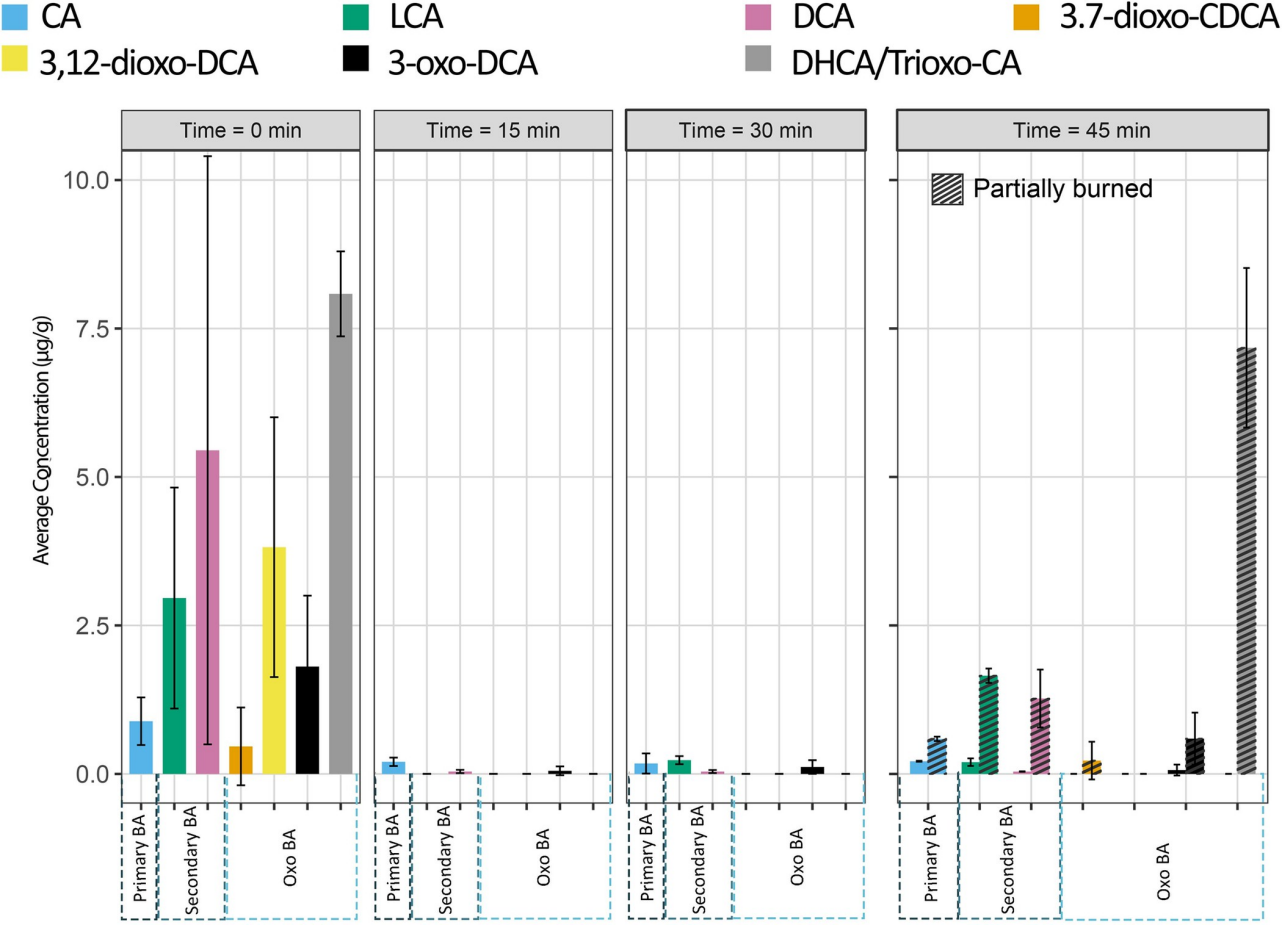
The concentration of bile acids extracted in control samples and samples burned in an open fire (data in S2 File). The partially and completely burned samples are displayed side by side for Time = 45 min.

**Fig 4:**
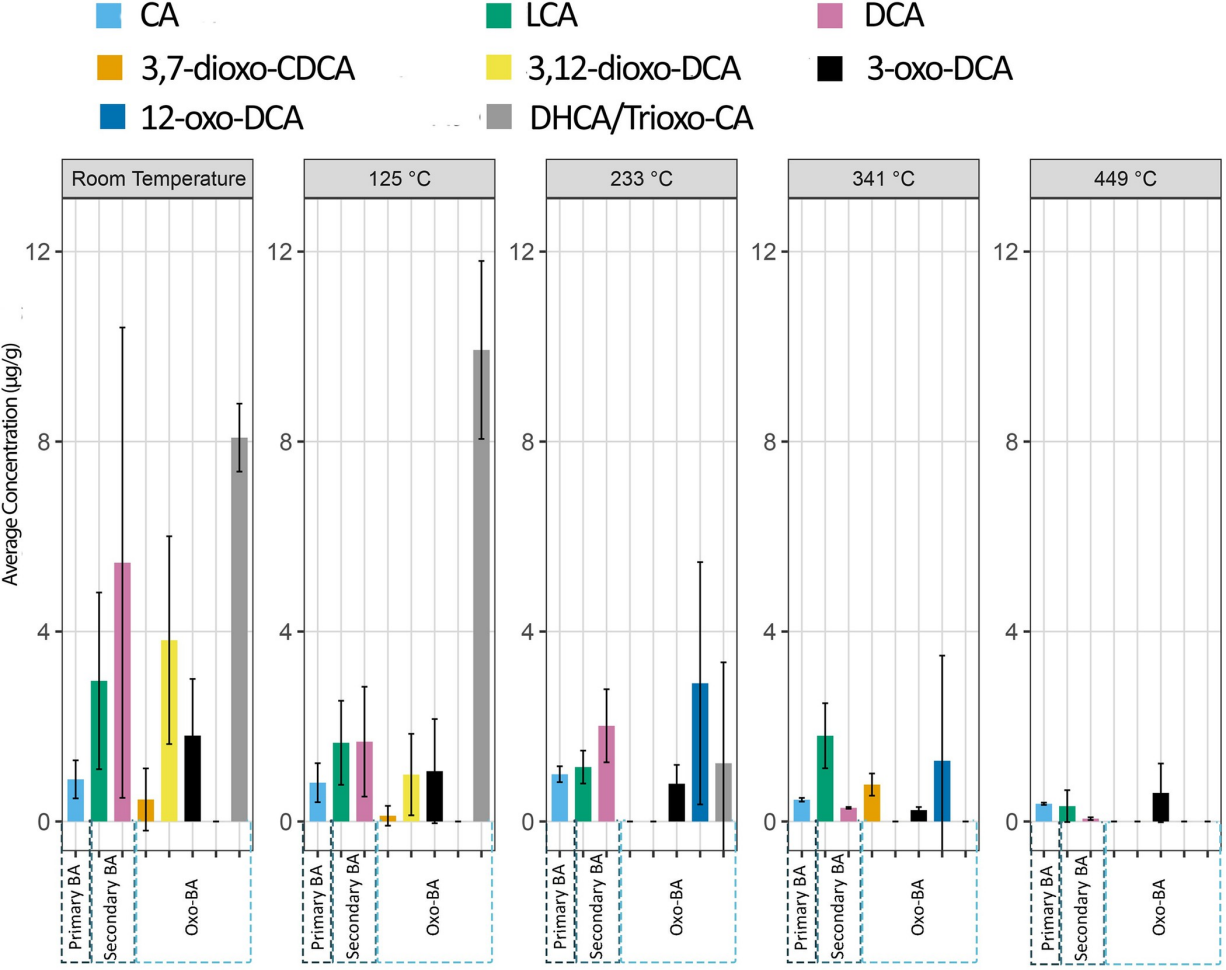
The concentration of bile acids extracted from control samples and samples burned in a muffle furnace with controlled temperature (data in S2 File).

All bile acid concentration was standardized against the TOC content of each dung sample. Our study shows that the control samples obtain primary, secondary, and oxo-bile acids (the full table with all bile acids and their concentration is in S2 File). Fig 3 illustrates that the only primary bile acid in our control cattle dung sample is cholic acid (CA), with a detected concentration range between 0.6–1.17 μg/g. However, as described earlier, this concentration is likely underestimated due to the lower recovery rate of CA, which was only 33%. Secondary acids included deoxycholic acid (DCA), ranging between 1.9–8.9 μg/g, and lithocholic acid (LCA), ranging between 1.6–4.3 μg/g. Interestingly, our study did not detect ursodeoxycholic acid (UDCA), a secondary bile acid that was previously reported by Sherina et al. (1968) and Porru et al. (2022) (Table 3). UDCA and 3-epideoxycholic acid (EDCA) are known to be produced in small amounts in ruminants [25]. Several oxo-bile acids were identified in our samples, including 12-oxo-DCA, 3,12-dioxo-DCA, 3,12-dioxo-DCA, DHCA /trioxo-CA, and 3,7-dioxo-CDCA. The most abundant oxo-bile acids in our control samples was dehydrocholic acid (DHCA/trioxo-CA), with a concentration ranging between 7.6–8.6 μg/g, a finding not reported in previous studies. However, considering the results of internal standards and probably underestimating the primary and secondary bile acids, there could also be biases for oxo-bile acids. The presence of 3,12-dioxo-DCA (2.3–5.4 μg/g) and 3-oxo DCA (0.9–2.6 μg/g) in our samples aligns with the findings of Porru and colleagues’ study [17]. Trace amounts of 3,7-dioxo-CDCA acid (0–0.9 μg/g) were also detected. In addition, 12-oxo-DCA, a bile acid known to be produced from cholic acids by intestinal microflora and present in low concentrations in cow feces [25], was identified in the control sample spectra. However, its concentration could not be accurately determined due to chromatographic overlap with another compound. Therefore, 12-oxo-DCA is not depicted in Figs 3 and 4 for the control samples but is evident in the burned samples, where the overlapping peak is absent. Furthermore, 3-oxo-LCA, 7-oxo-CDCA, and 3-oxo-CA were detected in our control samples. However, their final concentrations could not be calculated because the standard of these three compounds was not injected for this study. Nevertheless, our instrument detected clear signals for these compounds in the control samples.

**Table 3:** Bile acids in cattle dung in this study and previous studies.

Bile acids/research	Abbreviation	Sherina et al. 1968	Prost et al. 2017	Porru et al. 2022	Current study
Cholic acid	CA	x		x	X
Deoxycholic acid	DCA	x	x	x	X
Lithocholic acid	LCA	x	x	x	X
Ursodeoxycholic acid	UDCA	x		x	
Isodeoxycholic acid	IDCA		x		
Isolithocholic acid	ILCA		x		
Chenodeoxycholic acid	CDCA	x			
Eepideoxycholic acid	EDCA	x			
3α-OH-12-O-Cholanoic acid	12-oxo-DCA	x		x	X
3α12α-diOH-7-O-Cholanoic acid	3,12-dioxo-DCA			x	X
12α-OH-3-O-Cholanoic acid	3-oxo-DCA			x	X
3α,7α -diOH-12-O-Cholanoic acid	12-oxo-CA	x		x	
3α12α -diOH-7-O-Cholanoic acid	7-oxo-CA	x			
Dehydrocholic acid	DHCA /trioxo-CA				X
3,7-diO-Cholanoic acid	3,7-dioxo-CDCA				X

### Open-air burning experiment

The results from the open-air burning experiment demonstrate that high-temperature can profoundly affect bile acid concentration in fecal matter (Fig 1 and S2 File). The highest temperature fire occurred at the start of the experiment, with the first set of samples collected at 15 minutes of dung burning, during which the temperature ranged between 442–740°C at the center of the fire pits. By the second set of samples at 30 minutes, the temperature slightly decreased to between 457–570°C, and by 45 minutes, it had further dropped to 230–466°C. The data presented in Fig 3 show that, despite varying time intervals, carbonized samples exhibited low concentrations of bile acids. The primary bile acid CA was present in all samples regardless of the length of burning time, demonstrating remarkable resistance to high temperatures, although its concentration was significantly decreased fourfold. However, LCA and DCA proved to be more susceptible, with significantly reduced concentrations compared to control samples. Surprisingly, LCA was not detected in samples collected at 15 minutes of burning but was still present in small amounts in samples taken at 30 and 45 minutes. Furthermore, one of the oxo-bile acids, 3-oxo-DCA, was identified alongside CA and DCA during all time intervals.

We observed higher bile acid concentrations in the incompletely burned samples at 45 min than in completely burned dung ashes (Fig 3). Additionally, our analysis revealed the presence of 3,7-dioxo-CDCA acid in addition to CA, DCA, LCA, and 3-oxo-DCA. Following the results from the open-air experiment, we conclude that time did not arise as a significant influencer on the bile acid concentration changes.

### Muffle furnace burning experiment

With a fixed burning time of 60 minutes in the muffle furnace, our analyses revealed a decline in the number and concentration of bile acids at higher temperatures compared to lower ones (S2 File and Fig 4). The MANOVA statistical analysis showed a significant difference in bile acid concentrations among different temperature groups for the muffle burned and control samples (Pillai’s trace = 3.41, F_20,32_ = 3.63, P = 0.002). However, as depicted in Fig 4, CA, LCA, DCA, and 3-oxo-DCA exhibited relatively higher resistance to temperature elevation than other bile acids, similar to results from the open-air experiment. These compounds were detected despite the temperature rise. The ANOVA test on the MANOVA outputs indicates that CA, LCA, DCA, and 3-oxo-DCA did not significantly differ across different temperature groups (p = 0.05, 0.07, 0.06, 0.28, respectively), despite the decreased concentrations (for more details S3 File). Notably, 3-oxo-DCA is particularly resistant to heat compared to all other oxo-bile acids, as it remains remaining present even at 449°C, although its concentration steadily decreased. This bile acid was also detected in the open-air burning experiment at temperatures up to 740°C. By contrast, 3,12-dioxo-DCA was less tolerant to heating with its concentration significantly descreased (ANOVA, p = 0.004), as it was not detected in samples burned at 233°C or higher temperatures. Similarly, DHCA showed intolerance to high temperatures, completely disppeared in samples burned at 341°C and 449°C (ANOVA, p<0.001).

We identified 12-oxo-DCA at 233°C and 341°C. In the control samples and at 125°C, this compound was present but was difficult to quantify due to chromatographic overlap with another unknown compound. However, as burning progressed, the unknown compound disappeared, allowing for the clear detection and quantification of 12-oxo-DCA. Notably, this compound is not preserved after treatment at 449°C.

## Discussion

### Temperature and time effect on bile acid preservation

This study is the first to investigate how burning affects bile acid preservation and concentration. Our findings demonstrate that several primary and secondary bile acids can withstand high temperatures, although, their concentrations decrease as the temperature increases. As expected, oxo-bile acids decompose more rapidly during burning, making them less heat tolerant than primary and secondary bile acids. At 125°C, oxo-bile acids experience a decline in concentration but remain detectable. However, at 233°C, 3,12-dioxo-DCA and 3,7-dioxo-CDCA are no longer detectable. Unexpectedly, 3,7-dioxo-CDCA reappears at 341°C. At 449°C, the highest temperature in the furnace experiment, CA, LCA, DCA, and 3-oxo-DCA remain detectable but at significantly reduced concentrations. Since the open-fire experiments exceeded 740°C, the concentrations of these bile acids were comparatively lower than in the furnace experiment. This experiment shows that time did not significantly influence the changes in bile acid concentration. The open-air burning experiment demonstrated that the highest temperature was recorded at the earlier stage of the burning with the temperature gradually decreasing over time. Our observations are corroborated by a field study from prehistoric sheepfold caves in Spain, which found that, while bile acid concentrations were low or absent in most of the burned layers, high concentrations of bile acids were still present in a few partially burned feces [20]. Similarly, our findings from the open-fire experiment confirm that incompletely burned dung, typically found at the periphery of a hearth, retains higher concentrations of bile acids than fully burned samples (Fig 3). Based on these findings, we propose that future archaeological field studies collect at least two samples from the same horizon of a hearth: one from the fire center and another from the edge.

### Are bile acids in burned ash diagnostic of animal species?

The burning experiment shows that, unlike in unburned archaeological contexts, the preserved bile acids in burned ash are insufficient for discriminating between animal species. In unburned dung, a high percentage of DCA typically indicates ruminant origin, and equal concentrations of DCA and LCA indicate monogastric origin [7, 11, 20], corroborating our findings. Prost et al. [12] suggested that DCA should be a few folds greater than LCA in cattle and sheep feces, whereas donkey feces contain nearly equal amounts of both. Similarly, human and horse feces also contain an equal concentration of DCA and LCA [12] (p.16). Our finding shows that although DCA and LCA are both relatively heat-resistant, DCA has a lower tolerance to high temperatures compared to LCA, resulting in a skewed and unrepresentative ratio. Specifically, higher temperatures lead to greater degradation of DCA than the LCA, making it difficult to accurately distinguish species based on the pre-burning ratios. For instance, we found the average pre-burning DCA: LCA was approximately 2:1, whereas the post-burning DCA:LCA ratio became 1:3 for the open fire (between 442°C and 740°C) and 1:5 for muffle burning at 449°C. These post-burning ratios differ from previously reported unburning ratios that distinguish species [12]. These findings suggest that data published inferring ruminants versus omnivores from bile acid ratios of burned samples may need revision, particularly for samples with high ash content [e.g. 20, 26]. Given these discrepancies, there is a critical need to reassess the methodologies underlying interpretations of bile acid ratios in archaeological contexts involving burned remains.

Caution is also needed when considering the concentration of primary and secondary bile acids, as their levels can vary significantly due to external factors, such as diet, diseases, and environmental conditions (discussion below). Therefore, primary bile acids may not be the most reliable for distinguishing species because different feeding modes (e.g. purified diet without roughage and semipurified diet) and digestion can significantly affect bile acid concentration, even among animals with similar digestive systems. Instead, bile acid profiles are better suited for identifying broad categories of digestive physiology, such as differentiating between ruminants and non-ruminants. Additionally, hyodeoxycholic and ursodeoxycholic acids, two secondary bile acids frequently found in pig feces, warrant consideration for their potential to distinguish omnivorous digestive patterns [17]. However, the extent of their stability or degradation during burning is currently unknown. This uncertainty highlights the need for further research to understand better how these bile acids react under high-temperature treatment and their potential as reliable indicators in carbonized contexts.

### Bile acids in burned archaeological context as indicators for dung fuel

Our results demonstrate that several bile acids can be preserved even after exposure to high temperatures and complete combustion, albeit with a drastic reduction in concentration. This suggests that identifying fecal biomarkers in ashy and carbonized contexts can complement other methods in pinpointing dung-burning activities. The presence of bile acids in ashes indicates that animal dung was likely used as a fuel source, a practice that has been documented as having been practiced for millennia through archaeobotanical evidence [1, 27–30]. Since organic matter is not always preserved, archaeobotanical criteria have been established to identify the presence of dung burning [1, 31, 32]. These criteria include the presence of wild herbaceous seeds consumed by herds, a low abundance of wood, prevalence seeds smaller than 2 mm, and a high level of seed and fruit fragmentation. The challenge lies in tracing the transition from wood burning to the integration of wood and animal dung, especially when samples do not meet archaeobotanical standards based on seed abundance and types. Despite the robust criteria for identifying dung burning in archaeobotany, the seasonality of plant assemblages can introduce bias; notably, herd animals consume seeds during a limited part of the year. Archaeobotany combined with bile acids can yield additional insights into the temporal presence of pastoralist camps. For example, when archaeobotanical evidence does not confirm dung burning, the presence of bile acids would allow scholars to hypothesize that the campsite was inhabited during the spring months. This is because animals would have consumed wild, non-seed-bearing herbaceous plants during the that season. Furthermore, several well-established archaeological methods, such as micro-morphological, genetic analsis, and intestinal parasite studies, can confidently identify dung [10, 33–35]. Among them, one of the most effective is the examination of spherulites in sediments [36]. However, it is important to note that the absence of spherulites does not definitively indicate the absence of dung, as various factors can affect their preservation [36–39]. Therefore, fecal biomarkers are a powerful complement toexisting proxies for identifying domestic fuel use, seasonality, and domestication practice.

### Bile acid profiles in controlled samples and baseline biases

A comparison of bile acid profiles in our control samples with those reported in previous studies reveals significant variability in baseline concentrations and composition across different digestive physiologies and feeding modes (Table 3). These discrepancies in bile acid profiles can arise from differences in extraction techniques, sources of extraction, and even variations in diet or animal health conditions. Sheriha et al. [25] found that cattle feces contains a mixture of hydroxy and keto 5β-chorionic acids substituted at different positions, identified 13 bile acids in feces and fetuses from cows on natural or highly purified diets. The predominant bile acids in cow dung were CA, DCA, and CDCA, with smaller amounts of LCA, UDCA, and EDCA. Due to methodological advances, more recent studies have expanded the list to detectable bile acids. Prost et al. [12] detected IDCA, ILCA, LCA, and DCA. Porru et al. [17] used high-resolution mass spectrometry to uncover oxo-bile acids, like 12-oxo-DCA, 3,12-dioxo-DCA, 3oxo-DCA, and 12oxo-CA.

The concentration of bile acids can vary depending on the extraction source, as documented by earlier studies comparing bile acids extracted from cow bile, feces, fetuses, and serum [25, 40]. This variability is evident in our study and other recent research. Supporting our findings, Prost et al. [12] and Porru et al. [17] also did not detect CDCA in their samples despite its known presence in cattle bile. This absence is not entirely unexpected, as CDCA typically appears only in low amounts within cattle dung and is more characteristic of chicken and duck [41]. Interestingly, an earlier study observed a relatively higher CDCA concentration than CA in cattle bile. This observation suggests CDCA may be less readily absorbed into the enterohepatic circulation in the intestine than CA [25]. These findings underscore the importance of considering the specific biological matrix, when analyzing concentrations and compositions, as variability between matrices can significantly affect the results and their interpretations.

In contrast to other studies, we did not detect 7-oxo-CA in our cattle samples. This disparity may arise from variations in forage quality and nutrient availability, which are instrumental factors in shaping microbial populations that influence the bile acid metabolism of dairy cows [42]. Sheriha and colleagues [25] found that ruminant animals on a natural diet in conducive surroundings develop an optimal microflora in their digestive tracts, leading to DCA as the principal metabolic byproduct from a microbial transformation of CA. However, under less favorable conditions, such as when animals transition to a highly purified diet without roughage, bacterial conversion of CA to 7-oxo-CA becomes more prominent. Regional disparities in nutrient availability play a pivotal role in shaping microbial populations with distinct functional capacities influencing the bile acid metabolism of dairy cows [43]. These dietary influences extend beyond the production of specific bile acids, as Lin et al. [43] observed a notable difference in intestinal bile acid concentration between grain-fed and forage-fed groups of dairy cows. Grain-fed cattle exhibited markedly higher CA levels than those on a forage-based diet, likely due to the accumulation of unconjugated bile acids.

Dehydrocholic acid (DHCA), a synthetic bile acid produced from the oxidation of CA, was unexpectedly found in our control samples, raising questions about its origin and implications. Among several therapeutic uses, DHCA is effective in treating acute biliary pancreatitis (ABP) by safeguarding the pancreas and reducing inflammation [44, 45]. Its presence in our samples raises the question of whether DHCA was administered medically to the cattle or introduced, either advertently or inadvertently, through lick stones or feed supplements [46]. However, we have been unable to confirm such usage of DHCA in our particular case or within the EU in general. We also note that no studies have investigated the chemical transformation and elimination of DHCA after its administration to cattle as a feed additive. This gap highlights a potential area for future research to explore the metabolic pathways and residual presence of synthetic bile acids in livestock.

Earlier studies based on Gas Chromatography (GC) and GC-MS mainly detected primary and secondary bile acids, possibly due to their lower sensitivity than more recent analytical techniques [47]. Moreover, GC and GC-MS have limitations restricting their broad applicability in bile acid analysis. These limitations include the extensive time required for sample preparation, which encompasses extraction, purification, hydrolysis, and derivatization, as well as the inaccurate identification of stereoisomerization forms of bile acids when using a single gas column [19]. For these reasons, LC-MS and its derivatives have become the mainstream technique for bile acid analysis today. Advanced LC-MS techniques can detect many minor compounds and conjugated bile acids previously undetectable (e.g., [17, 48]). In addition to the choice of analytical technique, the extraction technique plays a crucial role in the accuracy and reproducibility of bile acid analysis results. For instance, Hubert et al. [48] demonstrated that applying four different extraction methods to human fecal samples yielded varying results and concentrations. This variability underscores the importance of carefully selecting and standardizing methods to ensure the accuracy and reproducibility of bile acid analyses across studies.

## Conclusion

We examined bile acid preservation in burned dung to assess its potential as an archaeological biomarker. Our findings reveal that high temperatures can drastically reduce concentrations of bile acids. Primary and secondary bile acids demonstrate a moderate tolerance to high temperatures compared to oxo-bile acids, with CA, DCA, and LCA exhibiting a relatively high tolerance to heat. Despite exposure to temperatures of 449°C in the furnace and even over 700°C in the open fire, CA, LCA, DCA, and 3-oxo-DCA were still preserved, albeit in diminished amounts. However, identifying the species origins of organic residues from burned contexts may be challenging. While the primary and secondary bile acids used for inferring species origins are preserved in the ash, their preservation ratios are likely skewed due to their different heat tolerances, making them unreliable for accurate biosynthesis sources identication. This finding highlights the importance of carefully interpreting bile acid ratios in the burned context. Despite these challenges, our study illustrates the efficacy of fecal biomarkers in assessing dung as a fuel source in archaeological studies

While not investigated in this study, we further acknowledge that animal diet, environmental conditions, and animal health can affect bile acid metabolism in gut microbes and, consequently, the concentration of bile acids in dung. Future dung-burning experiments involving diverse species across different regions and diets are essential to establish a robust and reliable baseline for archaeological studies or to alert researchers to potential biases. Controlled experimental and ethno-archaeological work will be important to improve interpretations of ancient dung utilization. In many parts of the world, today and throughout history, dung has been one of the most important pastoralist products, enabling human expansion to wood-poor environments despite cold climates and limited resources. While dung is known to serve as fuel, manure, and building material, further investigations into its diverse purpose can offer deeper insights into human activities, social dynamics, and cultural practices.

## Supporting information

S1 File Recovery percentage. (XLSX)

S2 File BA concentrations. (XLSX)

S3 File Two-way Multivariate Analysis of Variance (MANOVA) and Analysis of Variance (ANOVA). (DOCX)
