# Supplementary material for: Bile acids as biomarkers in carbonized archaeological sediment: Insights from dung burning experiments
Source: PLoS One. 2025 Feb 21;20(2):e0312699. doi: 10.1371/journal.pone.0312699 (PMC11845037; doi:10.1371/journal.pone.0312699)
Supplement: S3 File — (DOCX) [file pone.0312699.s003.docx]

**Bile acids as biomarkers in carbonized archaeological sediment:**

**Insights from dung burning experiments**

Basira Mir-Makhamad, Thomas Larsen, Daniel Giddings Vassao, Robert Spengler, and

Yiming V Wang

**Supplementary Table 3.** Two-way Multivariate Analysis of Variance (MANOVA) tests

showing different bile acids concentrations in dung after muffle burning experiment

influenced by temperature ((Pillai’s Trace=3.4123, F_32,20_=3.6289.8, P<<0.001). Analysis of

Variance (ANOVA) was then performed on the MANOVA output to assess which bile acids were more sensitive to temperature. Our ANOVA results show that 3,7-dioxo-CDCA,

3,12-dioxo-DCA, and DHCA were significantly influenced by temperature (p<0.005).

**MANOVA summary**

**___________________________________________________________________________**

Df Pillai approx F num Df den Df Pr(>F)

Temperature 4 3.4123 3.6289 32 20 0.001825 **

Residuals 9

__________________________________________________________________________

**ANOVA summary**

**__________________________________________________________________________**

Response LCA :

Df Sum Sq Mean Sq F value Pr(>F)

Temperature 4 9.1478 2.28695 3.2008 0.06794 .

Residuals 9 6.4303 0.71448

Response 3,7-dioxo-CDCA :

Df Sum Sq Mean Sq F value Pr(>F)

Temperature 4 1.34870 0.33717 4.8496 0.02314 *

Residuals 9 0.62574 0.06953

Response 3,12-dioxo-DCA :

Df Sum Sq Mean Sq F value Pr(>F)

Temperature 4 24.0574 6.0144 8.652 0.003768 **

Residuals 9 6.2563 0.6951

Response 3-oxo-DCA :

Df Sum Sq Mean Sq F value Pr(>F)

Temperature 4 3.2753 0.81882 1.4983 0.2815

Residuals 9 4.9184 0.54649

Response 12-oxo-DCA :

Df Sum Sq Mean Sq F value Pr(>F)

Temperature 4 19.043 4.7608 1.8772 0.1987

Residuals 9 22.825 2.5362

Response DCA :

Df Sum Sq Mean Sq F value Pr(>F)

Temperature 4 42.428 10.6071 3.3634 0.06041 .

Residuals 9 28.384 3.1537

Response DHCA :

Df Sum Sq Mean Sq F value Pr(>F)

Temperature 4 254.965 63.741 34.648 1.784e-05 ***

Residuals 9 16.557 1.840

Response CA :

Df Sum Sq Mean Sq F value Pr(>F)

Temperature 4 0.86633 0.216583 3.4975 0.05493 .

Residuals 9 0.55732 0.061925

**__________________________________________________________________________________**
